# Development of a disease-specific graded prognostic assessment index for the management of sarcoma patients with brain metastases (Sarcoma-GPA)

**DOI:** 10.1186/s12885-020-6548-6

**Published:** 2020-02-12

**Authors:** Anna Patrikidou, Loic Chaigneau, Nicolas Isambert, Kyriaki Kitikidou, Ryan Shanley, Isabelle Ray-Coquard, Thibaud Valentin, Bettina Malivoir, Maryline Laigre, Jacques-Olivier Bay, Laurence Moureau-Zabotto, Emmanuelle Bompas, Sophie Piperno-Neumann, Nicolas Penel, Thierry Alcindor, Cécile Guillemet, Florence Duffaud, Anne Hügli, Cécile Le Pechoux, Frédéric Dhermain, Jean-Yves Blay, Paul W. Sperduto, Axel Le Cesne

**Affiliations:** 1 Gustave Roussy Cancer Campus, Villejuif, France; 2 Present Address: Sarah Cannon Research Institute and UCL Cancer Institute & University College London Hospitals, 93 Harley Street, London W1G 6AD, UK; 3 IRFC, Besançon, France; 4 Centre François Leclerc, Dijon, France; 5 Democritus University, Orestiada, Greece; 6 Gamma Knife Center, University of Minnesota, Minneapolis, MN USA; 7 Centre Léon Bérard, Lyon, France; 8 Institut Claudius Regaud, Toulouse, France; 9 CHU Tours, Tours, France; 10 ICM, Montpellier, France; 11 CHU Clermont-Ferrand, Clermont-Ferrand, France; 12 Institut Paoli Calmettes, Marseille, France; 13 Centre René-Gauducheau, Nantes, France; 14 Institut Marie Curie, Paris, France; 15 Centre Oscar Lambret, Lille, France; 16 McGill University Health Centre, Montreal, Canada; 17 Centre Henri Becquerel, Rouen, France; 18 Hôpital La Timone, Marseille, France; 19 Geneva, Switzerland

**Keywords:** Sarcoma, Brain metastasis, Prognostic index

## Abstract

**Abstract:**

**Background:**

Brain metastases from sarcomatous lesions pose a management challenge owing to their rarity and the histopathological heterogeneity. Prognostic indices such as the Graded Prognostic Assessment (GPA) index have been developed for several primary tumour types presenting with brain metastases (e.g. lung, breast, melanoma), tailored to the specifics of different primary histologies and molecular profiles. Thus far, a prognostic index to direct treatment decisions is lacking for adult sarcoma patients with brain metastases.

**Methods:**

We performed a multicentre analysis of a national group of expert sarcoma tertiary centres (French Sarcoma Group, GSF-GETO) with the participation of one Canadian and one Swiss centre. The study cohort included adult patients with a diagnosis of a bone or soft tissue sarcoma presenting parenchymal or meningeal brain metastases, managed between January 1992 and March 2012. We assessed the validity of the original GPA index in this patient population and developed a disease-specific Sarcoma-GPA index.

**Results:**

The original GPA index is not prognostic for sarcoma brain metastasis patients. We have developed a dedicated Sarcoma-GPA index that identifies a sub-group of patients with particularly favourable prognosis based on histology, number of brain lesions and performance status.

**Conclusions:**

The Sarcoma-GPA index provides a novel tool for sarcoma oncologists to guide clinical decision-making and outcomes research.

## Background

Brain metastasis (BM) in adult sarcoma patients is a rare occurrence [1–3]. Owing to this rarity, little formal exploration exists in the literature, and evidence-based data is scant. In contrast, BM management in other cancer types has recently evolved in part due to advances in imaging and treatment but also because of the progressive development of prognostic indices.

Whereas in the past, it was believed that all patients with brain metastases had a grim prognosis, we now know that this patient population is markedly heterogeneous and prognosis varies widely. A number of prognostic indices were developed in order to guide treatment decisions, notably the RTOG (Radiation Therapy Oncology Group) Recursive Partitioning Analysis (RPA) [4, 5], the Score Index for radiotherapy (SIR) [6] and the Basic Score for Brain Metastases (BSBM) [7]. A more recent index, the Graded Prognostic Assessment (GPA) index [8], Table 1) was developed to address the limitations of previous indices, utilizing knowledge on the prognostic value of the number of brain metastases and shaping the index so as to guide treatment decisions rather than to reflect treatment results. Comparison with previous indices has indicated its improved utility and prognostic power [8]. The original GPA was validated and refined with disease-specific prognostic indices for the major types of cancer that develop brain metastases, such as breast, lung, melanoma, renal and gastrointestinal cancer [9, 10] and has evolved to incorporate information on histotype [11, 12] and tumour molecular characteristics [13, 14]. Ρopulation-based reports have confirmed the prognostic significance of histotype in breast cancer for the predilection of site of distant metastasis and the development of brain metastases [15, 16].

**Table 1:** Original Graded Prognostic Assessment (GPA) score

	Graded Prognostic Assessment (GPA) Scoring Criteria^a^		
Prognostic Factor	0	0.5	1.0
Age, years	> 60	50–60	< 50
KPS	< 70	70–80	90–100
ECM	Present	–	Absent
Number of BM	> 3	2–3	1

*KPS* Karnofsky performance status, *ECM* extracranial metastasis, *BM* brain metastases

^a^As per Sperduto et al 2008 Int J Radiation Oncol Biol Phys

Prognosis of brain metastases is not uniform throughout the different forms of cancer, nor amongst patients suffering from the same cancer type. This knowledge also implies that use of the same treatment for all patients and all primary types for the management of brain metastases is not appropriate, especially in the face of recent developments of treatment modalities.

The one-size-fits-all treatment paradigm that is no longer appropriate in other cancer types is still dominating the management of sarcoma patients with brain metastases.

Brain metastases in sarcoma patients is rather rare, with a reported incidence of < 1 to 8%. The French Sarcoma Group (GSF-GETO) has recently published the largest series to date of sarcoma patients with brain metastases, describing their characteristics, treatment modalities, prognostic factors and outcome [17]. This report identified leiomyosarcoma and liposarcoma as the most frequent histologies in sarcoma BM patients, and identified several characteristics of long survivors (younger age, unique lesions, lower grade tumors, better PS, longer time to development of brain metastases, higher use of local treatment modalities) [17].

On the basis of this cohort, we aimed to (a) assess the validity of the original GPA index in sarcoma patients with brain metastases, and (b) develop an informative, sarcoma-specific GPA index (Sarcoma-GPA), to serve as a prognostic index for treatment decisions and outcomes analyses.

## Methods

### Patient cohort and data collection

Under the auspices of GSF-GETO, a project involving a multi-institutional retrospective analysis project of sarcoma patients with brain metastases (cerebral or meningeal lesions) was developed (BRAINSARC) [17]. Institutional ethics committee approval was obtained for each centre. The database included patients from 15 French, one Swiss and one Canadian centre. The retrospective data collection was limited to patients managed between January 1992 and March 2012, to ascertain homogeneity in histological diagnosis and classification, namely uniform use of the FNCLCC grading system [18], and to ensure adequate follow-up. The results of this analysis are published elsewhere [17]. Utilizing and enriching this GSF-GETO database, we developed the current project of implementation of the original GPA on sarcoma patients and development of a disease-specific index (Sarcoma-GPA).

Data collection procedures for the BRAINSARC project are described in detail elsewhere [17]. Specifically for the current project, data collection was completed, verified and annotated for the GPA components, notably age at BM diagnosis, Karnofsky performance status (KPS), number of brain lesions and presence of extracranial metastases (ECM), as well as for overall survival (OS). For the development of the disease-specific GPA index, data on ECOG performance status, localization of brain metastasis, time to brain metastasis (TTBM), site of ECM, histological subtype and grade were also collected, verified and annotated. For the histological classification, the 2013 WHO Classification of Tumors of Soft Tissue and Bone was used [19].

### Statistical analysis

Overall survival was estimated from the time of BM diagnosis to the date of death or last follow-up. TTBM was estimated from initial sarcoma diagnosis to the time of BM diagnosis.

For the implementation of the GPA index on our sarcoma cohort, data for each of the four index components were coded according to the original GPA score [8] (Table 1). Each patient was attributed an overall score corresponding to the sum of the scores of individual index components. The GPA index was analyzed in four levels, as per the original description, with group cut-offs of 0–1, 1.5–2.5, 3 and 3.5–4. The GPA scores were subsequently correlated with OS. Survival distributions for individual variables but also for each individual index level compared with all other levels were compared with the log-rank and Mann-Whitney tests using a significance level of 0.001. Overall survival curves for each level of the GPA index were estimated using the Kaplan-Meier method, using the same significance level.

For the development of the Sarcoma-GPA index, cut-offs were decided based on previous GPA indices and on biological sense. Given that the study aim was to identify a meaningful, prognostic way of separating patient subgroups in terms of prognosis, in some instances different variations of cut-offs were attempted in order to identify significant, meaningful cut-offs. Prognostic factors for survival were analyzed by two methods: multivariate Cox regression (MCR) and recursive partitioning analysis (RPA). RPA aided in the identification of best splitting rules amongst prognostic factors. This dual MCR-RPA methodology has been previously shown to be an effective tool in the design of prognostic indices [10, 11, 20]. Prognostic factors found to be significant by either method were used to develop and refine the final Sarcoma-GPA index. Optimal cut-offs for groups were chosen to be consistent with previous disease-specific GPA literature (group cut-offs 0–1, 1.5–2, 2.5–3 and 3.5–4), weighing the significant factors in proportion to the magnitude of the hazard ratio such that 4.0 is the best and 0.0 is the worst [6, 10, 11, 13, 14]. Multivariate analysis was performed using the Cox proportional hazards model. Analyzed variables were age, KPS, ECOG PS, sarcoma type (bone versus soft tissue), localization, tumor size, histological grade and type, time to first metastasis, time to brain metastasis, TTBM, BM lesion number and localisation, presence of and type of ECM at the time of BM diagnosis, and all possible two-way interactions. For hazard ratios, the reference category is defined to have a HR = 1, HR > 1 indicating a higher death rate compared to the reference category. The univariate and multivariate analyses were performed separately for the ECOG PS and KPS variables, as these represent the same clinical characteristic (patient general functional status), in an attempt to identify any clinically pertinent difference in their use within the prognostic score. Since the objective was to develop a prognostic index to guide treatment, no treatment-related variable was analyzed. A forward selection procedure with a cutoff *p*-value of 0.05 was used to establish the initial model.

For the development of the final model, if individual classes within the investigated variables failed to show statistically significant differences of survival, groupings of multiple levels with similar outcomes were explored. Prognostic factors found to be significant by either MCR or RPA were retained in the final MCR model in order to improve its prognostic ability.

In the final Sarcoma-GPA index, a score of 4 correlates with the best prognosis and a score of 0 with the worst. The Kaplan-Meier method was used to estimate the survival curve for each prognostic group. The log-rank and Mann-Whitney test for censored data were used to test for significant survival differences amongst levels of the Sarcoma-GPA index (statistical significance defined as *p* < 0.001). The goodness of fit was evaluated using the Harrell’s concordance index (c-index), using 200 bootstrap replications to estimate out-of-sample performance, as well as ROC (Receiver Operating Characteristic) analysis. The final Sarcoma-GPA index was chosen as a balance of performance metrics and simplicity.

Analysis was performed using the SPSS Statistics version 22 (IBM Corp©, 2013).

The development of the sarcoma-specific index was done in collaboration with the team that described the original and disease-specific GPA indices.

## Results

A total of 251 patients with BMs (parenchymal, meningeal and combination of such lesions) of a sarcoma primary fulfilling the study criteria were included in the final analysis (5 patients that were excluded from the initially reported analysis owing to missing data were included in the current analysis as data were retrieved through a second, project-specific data collection as described above). The patient and disease characteristics are shown in Table 2, consistent to what has been previously reported [8]. Median follow-up was 2.79 months (OS range: 0.06–133.02 months). The median overall survival was 3.160 months. Presence of ECM was predominant at the time of BM diagnosis (91%), median TTBM was 18.5 months, whilst median time from first metastasis to development of BM (TMtBM) was 9.6 months. Treatment modalities details are presented in Additional file 1: Table S1.

**Table 2:** Cohort characteristics.

*Age*		*Primary tumor localization* (n, %)		*Number of lesions* (n, %)	
Median (yrs)	49.2	Limb	126 (50.2)	1	114 (45.4)
Range	16.1–85.6	Trunk/spine	43 (17.1)	2	45 (17.9)
(n, %)		Retroperitoneum	15 (6.0)	3	27 (10.8)
< 45	111 (44.2)	Uterus	15 (6.0)	4	14 (5.6)
45–55	44 (17.5)	Other	47 (18.7)	5	7 (2.8)
56–60	28 (11.2)	Unknown	5 (2.0)	> 5	41 (16.3)
> 60	68 (27.1)			Unknown	3 (1.2)
*Gender* (n, %)		*Initial tumor size* (cm)		*TTBM* (mo)	
Male	142 (56.6)	Median	9	Median	18.48
Female	109 (43.4)	Range	1–36	Range	0–215.34
*ECOG Performance Status (PS)* (n, %)		*Karnofsky Performance Status (KPS)* (n. %)		*BM localization* (n, %)	
0	47 (18.7)	90–100	67 (26.7)	Supra-tentorial	122 (48.6)
1	69 (27.5)	70–80	77 (30.7)	Infra-tentorial	12 (4.8)
2	56 (22.3)	50–60	57 (22.7)	Both	71 (28.3)
3	51 (20.3)	< 40	39 (15.5)		
4	17 (6.8)	Unknown	11 (4.4)	Meningeal	24 (9.5)
Unknown	11 (4.4)			Meningeal & parenchymal	16 (6.4)
				Unknown	6 (2.4)
*Type of sarcoma* (n, %)		*Extracranial metastasis* (n, %)		*TMtBM* (mo)	
Soft tissue	207 (82.47)	No	23 (9.2)	Median	9.6
Bone	44 (17.53)	Yes	228 (90.8)	Range	0–110.8
*Grade* (n. %)		Lung	184 (73.3)	*Chemotherapy lines prior to BM* (n, %)	
1	7 (2.8)	Liver	36 (14.3)	Median	1
2	41 (16.4)	Bone	62 (24.7)	Range	0–7
3	118 (47.0)	Other	76 (30.3)	At least 1 line	185 (73.7)
Unknown	85 (33.8)				
*Histology* (n, %)		*WBRT* (n, %)		*Intrathecal chemotherapy* (n, %)	
Leiomyosarcoma	46 (18.3)	Yes	147 (58.6)	Yes	3 (1.2)
Ewing/PNET	30 (12.0)	No	99 (39.4)	No	242 (96.4)
Liposarcoma	19 (7.6)	Unknown	5 (2.0)	Unknown	6 (2.4)
ASPS	14 (5.6)	*SRS* (n, %)		*Best response to BM treatment* (n, %)	
Osteosarcoma	14 (5.6)	Yes	24 (9.6)	CR	18 (7.2)
Rhabdomyosarcoma	14 (5.6)	No	221 (88.0)	PR	31 (12.3)
Angiosarcoma	14 (5.6)	Unknown	6 (2.4)	SD	64 (25.5)
Synovialosarcoma	13 (5.1)			PD	119 (47.4)
Uncertain differentiation/Other	82 (32.6)			Unknown/not evaluable	19 (7.6)
Unknown	5 (2.0)				
*Chemotherapy for BM* (n, %)		*Surgery* (n, %)		*Follow-up (mo)*	
Yes	91 (36.2)	Yes	39 (15.5)	Median	2.7
No	154 (61.4)	No	206 (82.1)	Range	0.1–133.0
Unknown	6 (2.4)	Unknown	6 (2.4)		

*ASPS* Alveolar soft part sarcoma, *BM* brain metastasis, *cm* centimeters, *CR* complete response, *ECOG* Eastern Cooperative Oncology Group, *KPS* Karnofsky performance status, *mo* months, *n* number, *OS* overall survival, *PD* progressive disease, *PNET* primitive neuroectodermal tumour**,** *PR* partial response, *PS* performance status, *SD* stable disease, *SRS* stereotactic radiosurgery, *TMtBM* time from first metastasis to brain metastasis, *TTBM* time to brain metastasis, *yrs*. years, *WBRT* whole brain radiotherapy

### Implementation of the original GPA score in sarcoma patients

The application of the original GPA score in our sarcoma patient cohort did not allow for validation of its prognostic value. The differences in median OS for each GPA index level were not significant for clear discrimination between each subgroup, especially for the higher-scoring subgroups (Additional file 3: Figure S1). Of the individual index components using score-specific cut-offs, the KPS was the most highly significant, showing the best discrimination amongst component levels (*p* < 0.001) (Additional file 4: Figure S2).

### Development of the sarcoma-GPA index

**Fig. 1:**
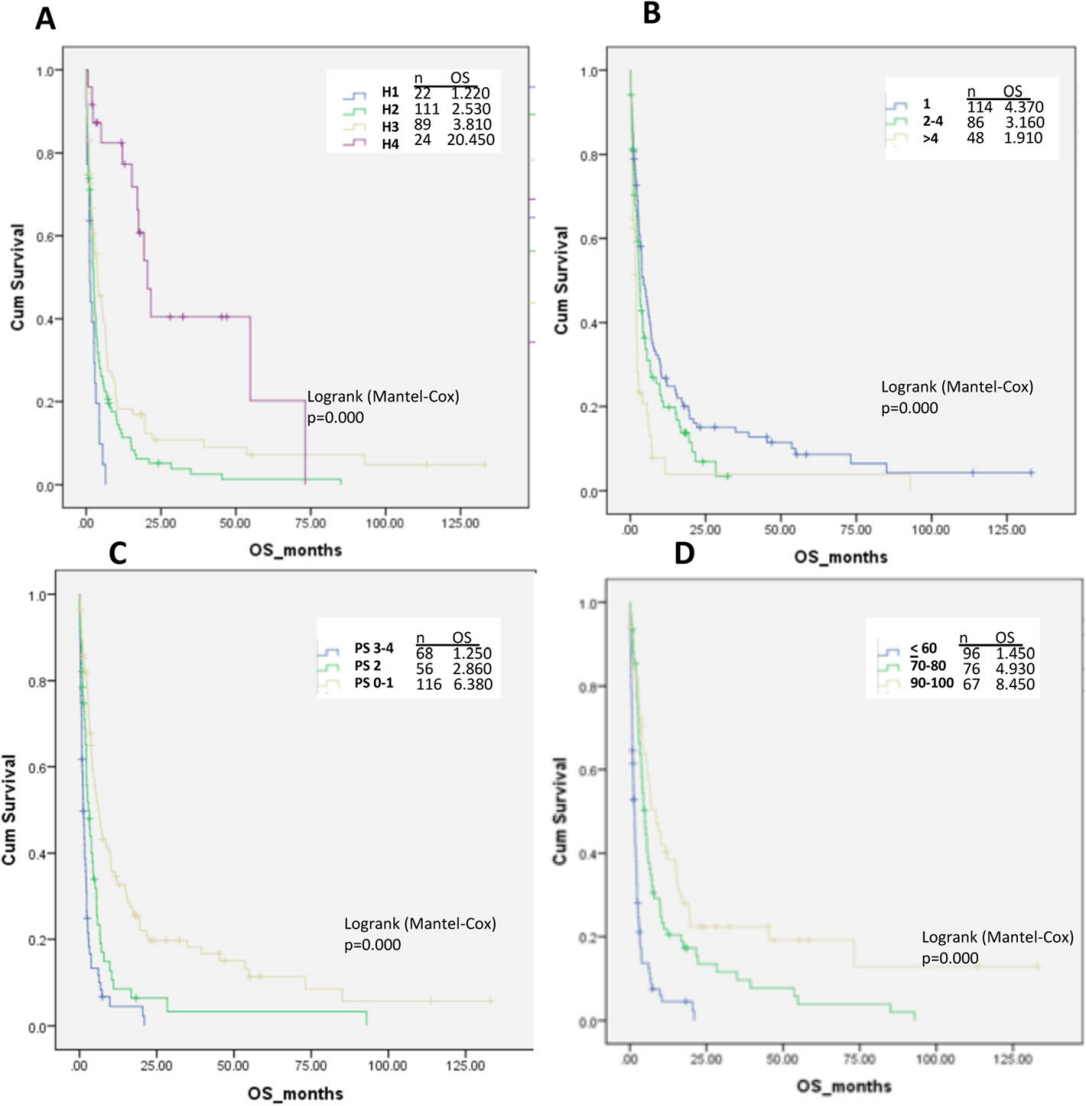
Significant variables in multivariate analysis. **a**: Histology; **b**: number of CNS metastases; **c**: ECOG performance status (PS); **d**: Karnofsky performance status (KPS). CNS: central nervous system; H1-H4: histology groups (see text for description); OS: overall survival

**Fig. 2:**
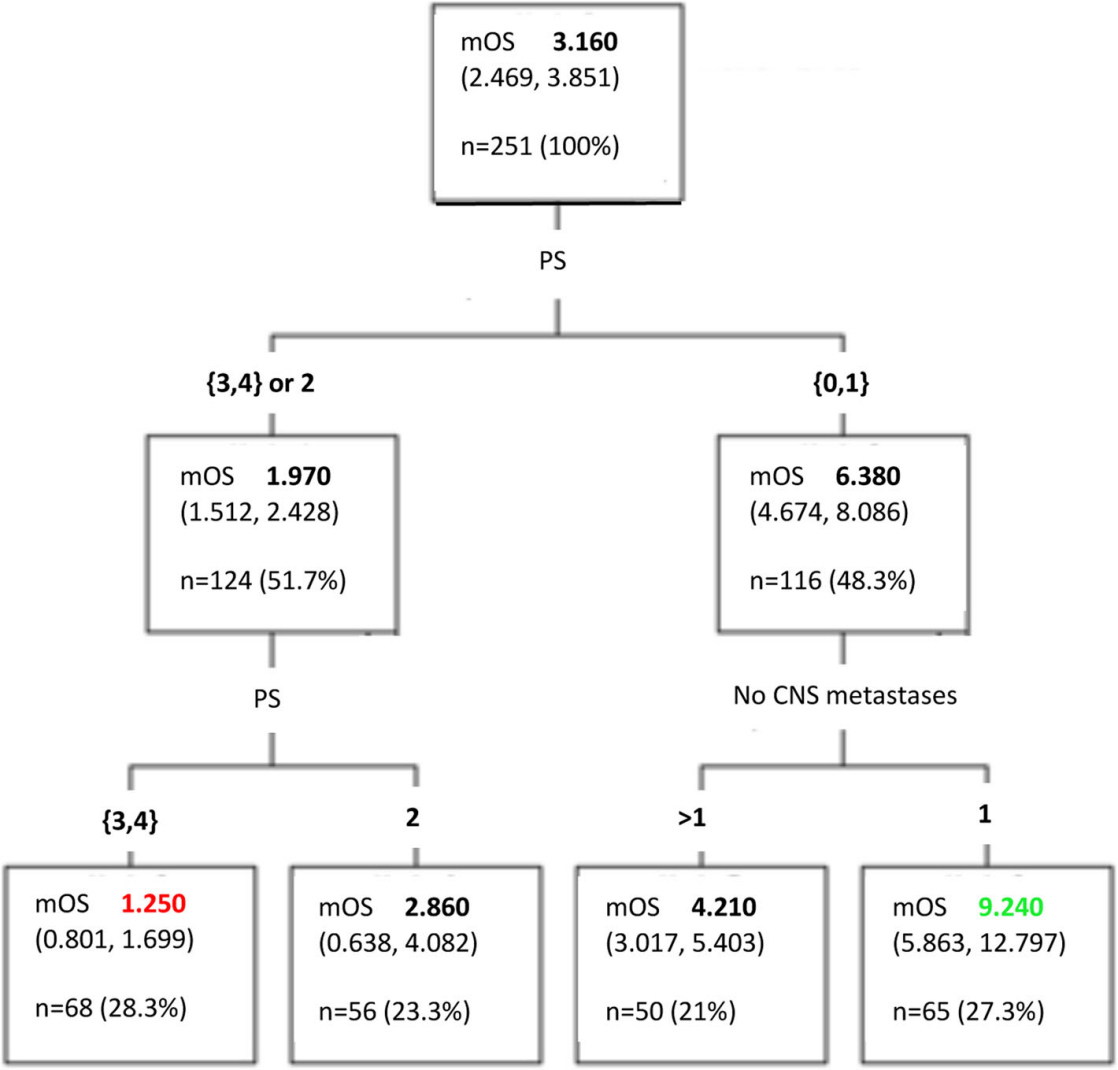
Recursive Partitioning Analysis (RPA) results. CNS: central nervous system; OS: overall survival; PS: performance status

Different variable cut-offs were individually assessed for significance in regard with overall survival, and were subsequently tested in the under development index. The variables identified as significant in the univariate (age, histology, number of CNS metastases, ECOG PS, KPS, TTBM) and multivariate analysis (histology, number of CNS metastases, ECOG PS, KPS) were individually assessed for the index (Fig. 1*,* Table 3). RPA analysis results were consistent with the MCR analysis, identifying the number of BMs and the ECOG PS as predictive for survival (Fig. 2).

**Table 3:** Univariate and multivariate analyses

Variable	UVA				MVA			
	*p*-value	HR	95% CI		*p*-value	HR	95% CI	
			Lower	Upper			Lower	Upper
Age (reference: > 55)								
Age [45–55]	.209	.765	.503	1.162	.099	.685	.436	1.074
Age < 45	**.003**	.646	.483	.863	.802	.957	.677	1.352
Gender	.620	.934	.714	1.222				
Sarcoma_type (bone vs soft tissue)	.701	.936	.667	1.312				
Histology (reference: H1)								
Histology: H2	**.016**	.557	.346	.896	**.006**	.493	.297	.819
Histology: H3	**.000**	.381	.231	.627	**.000**	.334	.192	.581
Histology: H4	**.000**	.129	.063	.263	**.000**	.159	.072	.349
Grade (reference: 3)								
Grade = 2	.157	.756	.513	1.114				
Grade = 1	.174	.561	.244	1.291				
Extracranial metastases (no, yes)	.679	.907	.571	1.441				
Extracranial metastases: Lung (no, yes)	.621	1.079	.798	1.458				
Extracranial metastases: Liver (no, yes)	.521	.881	.598	1.297				
Extracranial metastases: Bone (no, yes)	.563	1.099	.798	1.513				
Extracranial metastases: other (no, yes)	.291	.853	.636	1.145				
Number of CNS metastases (reference: > 4)								
Number of CNS metastases (2–4)	**.007**	.590	.402	.866	.092	.699	.462	1.060
Number of CNS metastases (1)	**.000**	.453	.314	.654	**.022**	.620	.412	.933
ECOG PS (reference: 3 or 4)								
ECOG PS = 2	**.001**	.523	.359	.761	**.000**	.468	.314	.696
PS (0 or 1)	**.000**	.274	.195	.384	**.000**	.305	.211	.441
Localisation (reference: supra-tentorial)								
Localisation: infra-tentorial	.317	.763	.450	1.295				
Localisation: supra-tentorial & infra-tentorial	.887	1.057	.490	2.282				
Localisation: meningeal	.120	1.555	.892	2.712				
Localisation: parenchymal & meningeal	.504	.801	.417	1.537				
TTBM (reference: < 12 months)								
TTBM (12–24 months)	.536	1.120	.782	1.604	.934	1.017	.686	1.507
TTBM (24–60 months)	.746	.944	.667	1.336	.461	.869	.598	1.263
TTBM > 60 months	**.000**	.453	.299	.686	**.006**	.526	.332	.834

*CI* confidence interval, *ECOG* Eastern Cooperative Oncology Group, *H1-H4* histology groups (see text for description), *HR* hazard ratio, *MVA* multivariate analysis, *No* number, *PS* performance status, *TTBM* time to brain metastasis (in months), *UVA* univariate analysis

Entries in bold represent statistically significant values

**Fig. 3:**
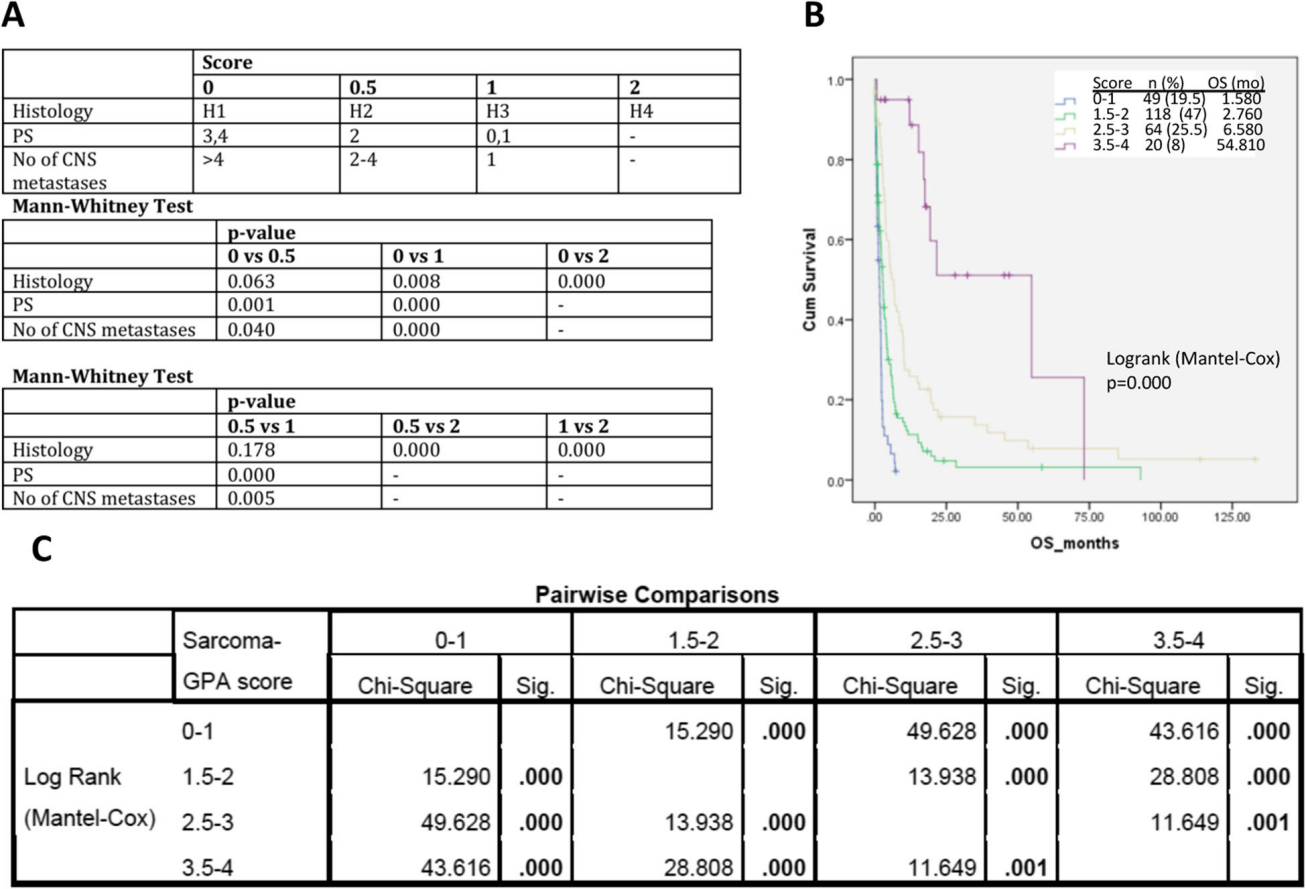
Sarcoma Graded Prognostic Assessment (Sarcoma-GPA) index. **a**. Prognostic factors, point groupings and Mann-Whitney test for significance of split levels; **b**. Kaplan-Meier curves for overall survival levels by Sarcoma-GPA group; **c**: Pairwise comparisons using the Mantel-Cox logrank test for the Sarcoma-GPA groups, demonstrating statistically significant separation between groups. CNS: central nervous system; H1-H4: histology groups (see text for description); OS: overall survival; PS: ECOG performance status

For the development of the sarcoma-specific GPA, the variables and respective cut-offs identified as significant were tested in different combinations. The best performing split levels and groups, as indicated by both MCR and RPA, lead to the identification of the optimal Sarcoma-GPA index, that included the three variables retained as significant: histology, number of CNS metastases and performance status (Fig. 3). The final index used 4-point cut-offs for the prognostic group levels (scores 0–1, 1.5–2.0, 2.5–3 and 3.5–4), consistent with previously reported GPA scores, with the GPA1 group (score 0–1) having the worst prognosis and the GPA4 group (score 3.5–4) having the better prognosis (Fig. 3). The spit levels chosen for the Sarcoma-GPA index for the histology variables were as follows: group H1 (*n* = 22; adipocytic tumors, including liposarcoma and myxoid liposarcoma), group H2 (*n* = 111; smooth muscle tumors including leiomyosarcoma; skeletal muscle tumours including rhabdomyosarcoma; chondro-osseous tumors including osteosarcoma; fibroblastic/myofibroblastic tumors including fibrosarcoma; so-called fibrohistiocytic tumors including pleiomorphic MFH”/ undifferentiated pleiomorphic sarcoma; “vascular tumors, including angiosarcoma; tumors of uncertain differentiation including intimal sarcoma), group H3 (*n* = 89; tumors of uncertain differentiation, including synovial sarcoma, clear cell sarcoma, epithelioid sarcoma, small round cell tumors, undifferentiated sarcomas, and also malignant peripheral nerve sheath tumor/neurofibrosarcoma and one case of phyllodes tumor/cystosarcoma of the breast) and group H4 [*n* = 24; predominantly alveolar soft part sarcomas (*n* = 14) and solitary fibrous tumors (SFT)/hemangiopericytoma (*n* = 7)], with H4 having the best prognosis and H1 the worst; all individual pairwise comparisons showed statistically significant difference. The split level chosen for ECOG PS were 0–1, 2 and 3–4. For the number of CNS metastases, the split levels found to be more informative were 1, 2–4 and > 4 lesions, differently to the original GPA score.

The log-rank test of the final model and all pairwise comparisons showed a statistically significant difference in median OS between each sarcoma-GPA grouping (*p* < 0.0001) (Fig. 1c). The addition of the variables age, TTBM and presence of ECM in the under construction indices, assessed at several different split-levels, did not improve their prognostic significant, but rather compromised it (data not shown).

Although both ECOG PS and KPS were individually prognostic (Fig. 2e*&f*), ECOG PS was found to have a better separation power amongst sub-groups within the final index in comparison to KPS (Fig. 3 vs Additional file 5: Figure S3). The c-index for the original GPA was 0.649, which improved to 0.688 using the Sarcoma-GPA. The ROC curves for the original GPA and Sarcoma GPA corroborated the c-index results (data not shown).

As the Sarcoma-GPA index was designed to provide prognostic information independently of treatment modality, the use of the treatment modalities in our patient cohort was assessed for statistically significant difference. Additional file 2: Table S2 shows the distribution of the different treatment modalities in the individual histology sub-groups as defined above for the Sarcoma-GPA index. No statistically significant difference was observed for any of the treatment modalities between histology groups, with the exception of targeted therapy.

We also assessed whether OS was significantly different in the better prognosis groups (H4 and GPA4) according to the different treatment modalities. The differences were not statistically significant, indicating that the use of different treatment modalities did not significantly influence outcome; for the H4 group the *p* values were 0.222, 0.386, 0.019, 0.061, 1.00 and 0.37, whilst for the GPA4 group the p values were 0.048, 0.125, 0.048, 0.245, 0.938 and 0.107 for the WBRT, SRS, surgery, systemic chemotherapy, targeted therapy and BSC, respectively (not applicable for the intrathecal chemotherapy, as no patient in the H4 or GPA4 groups received this modality). Significance cut-off for the above was the same used for the construction of the Sarcoma-GPA index, i.e. *p* < 0.001).

## Discussion

Two components of the original GPA index were retained in the Sarcoma-GPA, albeit modified. Notably, the patient general status was included in the final index scored according to the ECOG PS score, as was more informative within the final index compared to KPS. The number of BMs was also retained, however alternative split-levels was found to be more informative within the final index (1 vs 2–4 vs > 4 lesions) (Fig. 3).

The presence of extracranial metastases, a component of the original GPA index [8] (Table 1), the lung cancer-specific GPA [10], and maintained in the updated Lung-molGPA [13], as well as in the Melanoma-molGPA [14], was not found to be prognostic for sarcoma BMs, potentially as an influence of the predominance of the presence of ECM at the time of sarcoma BM diagnosis, a finding consistent with previous literature [3]. Similarly, age, another original GPA index variable, was not retained as significant despite repeated analyses at different split-levels.

The important addition of the histology in the Sarcoma-GPA has helped increase the discriminative power of the index and identify a histology subgroup with especially good prognosis (the H4 histology group, median OS 20.45 months). The final combined index is able to stratify patients with a OS of more or less than 6 months, with two sub-groups on either side of this timepoint. The discriminative power of histological type is not surprising for sarcomas, as they comprise a highly diverse tumour group, with distinctive pathological features and molecular basis, as well as variable prognosis. In this context, the Sarcoma-GPA index described here is akin to the updated molecular GPA indices for lung cancer and melanoma [13, 14]. In the Sarcoma-GPA, the combination of the H4 histology groups tumors with a limited number of BMs and a good ECOG performance status is able to select for a particularly favorable prognosis group, with an estimated median OS of almost 55 months (Fig. 3).

ASPS is a rare histology, characterised by a specific molecular change [t(X;17)(p11;q25) translocation, resulting in an ASPL-TFE3 gene fusion] [21], and is known to have an indolent clinical course in the non-metastatic stage, however characterized by late metastases with a 5-year OS of 20% at the metastatic stage [22, 23]. ASPS feature a well-established preponderance for BMs, with a reported incidence of approximately 20–35% [22, 24–28], compared to < 1–8% of sarcoma patients developing BMs overall [3, 29, 30]. Our study featured a median OS for the ASPS cohort (*n* = 14) of 17.33 months, indicating that a relatively long survival is retained event in the presence of BMs, consistent with previous sporadic reports [27]. In contrast to traditional reports of ASPS as frequently associated with ECM [22, 24], none of our 14 cases were; this might be due to routine brain staging of asymptomatic patients at diagnosis, and also explain the relatively long survival, as these patients had relatively low-volume metastatic disease (~ 70% had a single BM, none had > 4 lesions, none featured ECM). Our haemangiopericytoma/SFT cases were similarly not associated with ECMs (although they were not primary intracranial meningeal haemangiopericytomas), consistent with the majority of previous literature, which nevertheless is extremely limited [31–34].

The value of our report is highlighted by the difficulty of obtaining large-volume data for BMs of sarcoma patients, given their rarity. The development of the BRAINSARC project was an optimal opportunity to develop a prognostic index for this heterogeneous group of diseases. Within the BRAINSARC project, we had identified a subset of patients with survival longer than 2 years [17]. Histology alone was not able to select for these patients as this group, other than ASPS and SFTs, also included leiomyosarcoma, synovialosarcomas and Ewing/PNET tumors. This is concurrent with our analysis, as the H4 histology group had a median OS of 20.45 months (Fig. 2), i.e. less than the > 24 months necessary to be classified as long survivors in our previous report. When, however, the H4 histology group variable was enriched within the overall index by its association with ECOG PS and number of BMs, this became a much more powerful prognostic tool (Fig. 3). Our previous long-survivor analysis, which indicated that long survivors featured a greater percentage of unique BM lesions and better ECOG PS, corroborates this [17].

It should be noted that the construction of the histology sub-groups was based on the split levels indicated by statistical analysis in regard with significantly differential survival, and not selected based on histological lineage, nevertheless a certain lineage coherence is indeed reflected in the H1-H4 grouping (for example, adipocytic tumors in H1 and musculoskeletal tumors in H2). Once the overall cohort was optimally split in the four survival groups, the histological types comprising these four subgroups were detailed, as described in the Results section. It is therefore a grouping pertaining to the survival of sarcoma BM patients, a meaningful way to segregate how different histologies fare according to BM patient survival, and not a strict histological affinity classification.

Although the choice of treatment modality is beyond the scope of this manuscript, it is important to highlight that his study reports on patients managed over a very large period of time, as was necessary in order to obtain a large enough cohort, owing to the rarity of brain metastases in sarcoma patients. In this period of over 25 years since the beginning of our reporting period, management of metastatic brain disease has enormously evolved, from very conservative and restricted to more aggressive even in the presence of extracranial disease, and this is reflected in the reported treatment modalities. Overall, the use of different treatment modalities, and notably local modalities known to be associated with better outcome in general (surgery, SRS) neither differed nor significantly influenced OS in the histology and GPA groups, which indicates that the better outcome of the H4 and GPA4 group was not influenced by a differential use of treatment modalities in these sub-groups, further strengthening the prognostic value of the index we describe in this paper.

The high spatial and temporal tumoral heterogeneity and clonal shift occurring between primary and metastatic sites poses a complex therapeutic challenge. Brain metastases are a distant reflection of the primary, with the specific peculiarities of the CNS microenvironment. The decision to apply a treatment or not in the presence of BMs is a cardinal one, and precedes the one of the modality choice. The aim of this paper was to derive a purely prognostic index, in order to provide guidance into the decision of treating a patient with sarcoma BM lesion(s). It is constructed on a smaller number of patients than the previous GPA indices, however this needs to be assessed in the context of the relative rarity of the sarcoma BMs. This index does not take into consideration the potential effects of treatment on the patient quality of life, a factor that needs to be evaluated for the final decision-making.

With the increased incidence of BMs in all cancer types and the evolvement of systemic treatment options leading to globally increased cancer survival, it has become crucial to adapt treatment attitudes for the presence of brain lesions, correctly identify patients that merit local treatment, obtain realistic estimates of survival and select for the optimal treatment strategy. This has been an issue ignored for long in the development of new strategies and drug development, but the paradigm has already started to change. Modern strategies for clinical trials not only allow and stratify for the presence of BMs, but trials are also specifically designed for BM patients. Even further, prognostic indices are nowadays incorporated in the design of clinical trials [35].

## Conclusions

The Sarcoma-GPA provides a novel tool to sarcoma oncologists to guide clinical decision-making and outcomes research. Tailored to the specifics of histological variations and characteristics of sarcoma patients with lesions homing to the brain, it identifies favorable prognosis patients that are more likely to gain an enhanced clinical benefit from BM-directed treatment. Prospective independent validation of the described index is needed, and this is currently planned in the context of a multinational project, as the rarity of sarcoma brain metastasis dictates the need for such a collaborative effort.

## Supplementary information

**Additional file 1: Table S1.** Treatment modalities. BSC: best supportive care; MD: missing data; SRS: stereotactic radiosurgery; WBRT: whole-brain radiotherapy

**Additional file 2: Table S2.** Treatment modalities per histology group BSC: best supportive care; SRS: stereotactic radiosurgery; WBRT: whole-brain radiotherapy.

**Additional file 3: Figure S1.** Application of the original GPA index in the sarcoma patient cohort. A. Prognostic factors, point groupings and Mann-Whitney test for significance of split levels; B. Kaplan-Meier curves for overall survival for the original GPA score; C: Pairwise comparisons for the original GPA index using the Mantel-Cox logrank test, demonstrating insufficient separation between groups in sarcoma patients. CNS: central nervous system; KPS: Karnofsky performance status; OS: overall.

**Additional file 4: Figure S2.** Original GPA components applied in our sarcoma cohort. A. Age; B. Karnofsky performance status (KPS); C. Number of CNS lesions; D. Presence of extracranial metastases. CNS: central nervous system; OS: overall survival.

**Additional file 5: Figure S3.** Sarcoma Graded Prognostic Assessment index based on KPS. A: Prognostic factors, point groupings and Mann-Whitney test for significance of split-levels; B: Kaplan-Meier curves for overall survival levels by Sarcoma-GPA group; C: Pairwise comparisons using the Mantel-Cox logrank test. H1-H4: histology groups (see text for description). CNS: central nervous system; KPS: Karnofsky performance status; OS: overall survival.

## Data Availability

Individual patient data are part of the individual centre clinical databases. Raw data supporting the findings can be requested by contacting the Corresponding author.

## Abbreviations

BM: Brain metastasis
BSSM: Basic Score for Brain Metastases
ECM: Extracranial metastases
FNCLCC: Fédération nationale des Centres de lutte contre le cancer
GPA: Graded Prognostic Assessment
GSF-GETO: Groupe Sarcomes Française - Groupe d’Etudes des Tumeurs Osseuses
KPS: Karnofsky performance status
RPA: Recursive Partitioning Analysis
RTOG: Radiation Therapy Oncology Group
