# Supplementary material for: Development of a disease-specific graded prognostic assessment index for the management of sarcoma patients with brain metastases (Sarcoma-GPA)
Source: BMC Cancer. 2020 Feb 12;20:117. doi: 10.1186/s12885-020-6548-6 (PMC7014599; doi:10.1186/s12885-020-6548-6)
Supplement: Supplementary file 1 — Additional file 1: Table S1. Treatment modalities. BSC: best supportive care; MD: missing data; SRS: stereotactic radiosurgery; WBRT: whole-brain radiotherapy [file 12885_2020_6548_MOESM1_ESM.docx]

| **Treatment modalities** | **Overall population N=251** | |
| --- | --- | --- |
|  | **n** | **%** |
|  |  |  |
| **Surgery** | 39 | 15.5 |
| Solitary lesion | 20 | 7.9 |
| Multiple lesions | 19 | 7.6 |
| Median OS (months) | 10.1 |  |
| Range [min - max] | [1.0 – 73.2] |  |
|  |  |  |
| **Radiotherapy** | 161 | 64.1 |
| WBRT | 137 | 54.6 |
| Median OS (months) | 3.9 |  |
| Range [min - max] | [0.2 – 133.0] |  |
| SRS | 19 | 7.6 |
| Median OS (months) | 5.6 |  |
| Range [min - max] | [0.9 – 92.9] |  |
| WBRT+SRS | 5 | 2.0 |
| Median OS (months) | 15.5 |  |
| Range [min - max] | [10.1 – 113.7] |  |
| MD | 5 | 2.0 |
|  |  |  |
| **Chemotherapy** | 95 | 37.8 |
| Median line | 1 |  |
| Range [min-max] | [1 - 2] |  |
| Median OS (months) | 7.7 |  |
| Range [min - max] | [6.2 – 11.8] |  |
| Combination regimen | 38 | 15.1 |
| Drugs |  |  |
| Doxorubicin | 23 | 9.2 |
| Ifosfamide | 22 | 8.8 |
| Etoposide | 20 | 8.0 |
| Trabectedin | 11 | 4.4 |
| Platinum agents | 10 | 4.0 |
|  |  |  |
| **Targeted therapy** | 11 | 4.4 |
| Median OS (months) | 15.5 |  |
| Range [min - max] | [1.7 - /] |  |
| Sorafenib | 5 | 2.0 |
| Sunitinib | 5 | 2.0 |
| Imatinib | 1 | 0.4 |
|  |  |  |
| **BSC alone** | 46 | 18.3 |
| Median OS (months) | 0.8 |  |
| Range [min - max] | [0.6 – 1.4] |  |
