# Supplementary material for: Development of a disease-specific graded prognostic assessment index for the management of sarcoma patients with brain metastases (Sarcoma-GPA)
Source: BMC Cancer. 2020 Feb 12;20:117. doi: 10.1186/s12885-020-6548-6 (PMC7014599; doi:10.1186/s12885-020-6548-6)
Supplement: Supplementary file 2 — Additional file 2: Table S2. Treatment modalities per histology group BSC: best supportive care; SRS: stereotactic radiosurgery; WBRT: whole-brain radiotherapy. [file 12885_2020_6548_MOESM2_ESM.docx]

|  | WBRT  (p=0.184) | | SRS  (p=0.044) | | Surgery  (p=0.030) | | Systemic chemotherapy  (p=0.007) | | Intrathecal  Chemotherapy  (p=0.761) | | Targeted  Therapy  (p=0.000) | | BSC  (p=0.005) | | |
| --- | --- | --- | --- | --- | --- | --- | --- | --- | --- | --- | --- | --- | --- | --- | --- |
|  | yes | no | yes | no | yes | no | yes | no | yes | no | yes | no | yes | no |  |
|  | (n,%) | | (n,%) | | (n,%) | | (n,%) | | (n,%) | | (n,%) | | (n,%) | |  |
| H1 | 15  (6.1) | 7  (2.9) | 20  (8.2) | 2  (0.8) | 17  (6.9) | 5  (2.0) | 19  (7.8) | 3  (1.2) | 22  (9.1) | 0  (0.0) | 22  (9.0) | 0  (0.0) | 13  (5.3) | 9  (3.7) |  |
| H2 | 41 (16.7) | 70 (28.6) | 104 (42.4) | 7  (2.9) | 100  (40.8) | 11  (4.5) | 74  (30.2) | 37  (15.1) | 108  (44.6) | 2  (0.8) | 110  (44.9) | 1  (0.4) | 89  (36.3) | 22  (9.0) |  |
| H3 | 34 (13.9) | 54 (22.0) | 79  (32.2) | 9  (3.7) | 76  (31.0) | 12  (4.9) | 47  (19.2) | 41  (16.7) | 85  (35.1) | 1  (0.4) | 84  (34.3) | 4  (1.6) | 76  (31.0) | 12  (4.9) |  |
| H4 | 9  (3.7) | 15  (6.1) | 18  (7.3) | 6  (2.4) | 13  (5.3) | 11  (4.5) | 14  (5.7) | 10  (4.1) | 24  (9.9) | 0  (0.0) | 18  (7.3) | 6  (2.4) | 22  (9.0) | 2  (0.8) |  |
