# Supplementary figures and images for: Development of a disease-specific graded prognostic assessment index for the management of sarcoma patients with brain metastases (Sarcoma-GPA)
Source: BMC Cancer. 2020 Feb 12;20:117. doi: 10.1186/s12885-020-6548-6 (PMC7014599; doi:10.1186/s12885-020-6548-6)

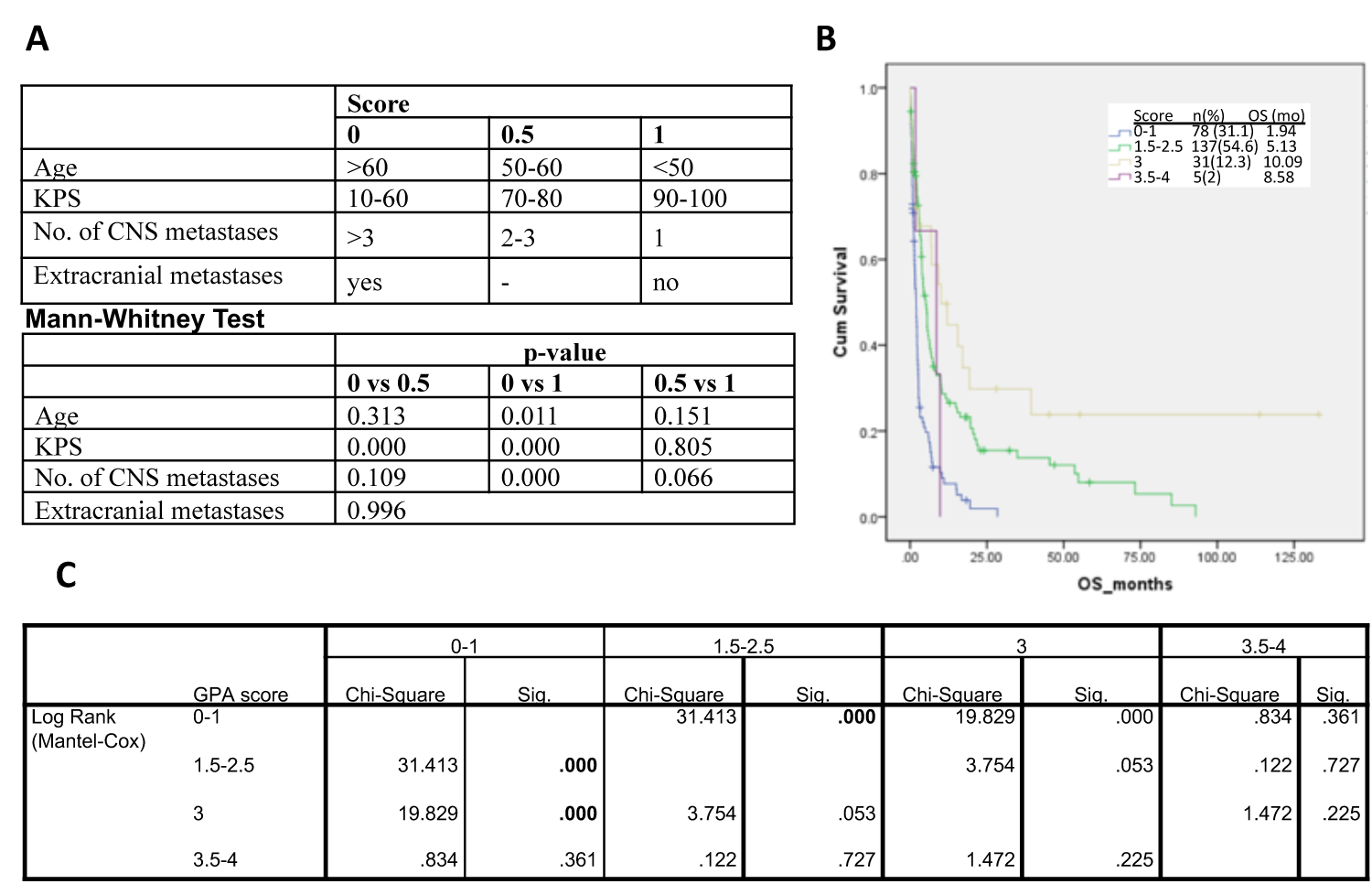

Supplement: Supplementary file 3 — Additional file 3: Figure S1. Application of the original GPA index in the sarcoma patient cohort. A. Prognostic factors, point groupings and Mann-Whitney test for significance of split levels; B. Kaplan-Meier curves for overall survival for the original GPA score; C: Pairwise comparisons for the original GPA index using the Mantel-Cox logrank test, demonstrating insufficient separation between groups in sarcoma patients. CNS: central nervous system; KPS: Karnofsky performance status; OS: overall. [file 12885_2020_6548_MOESM3_ESM.tif]

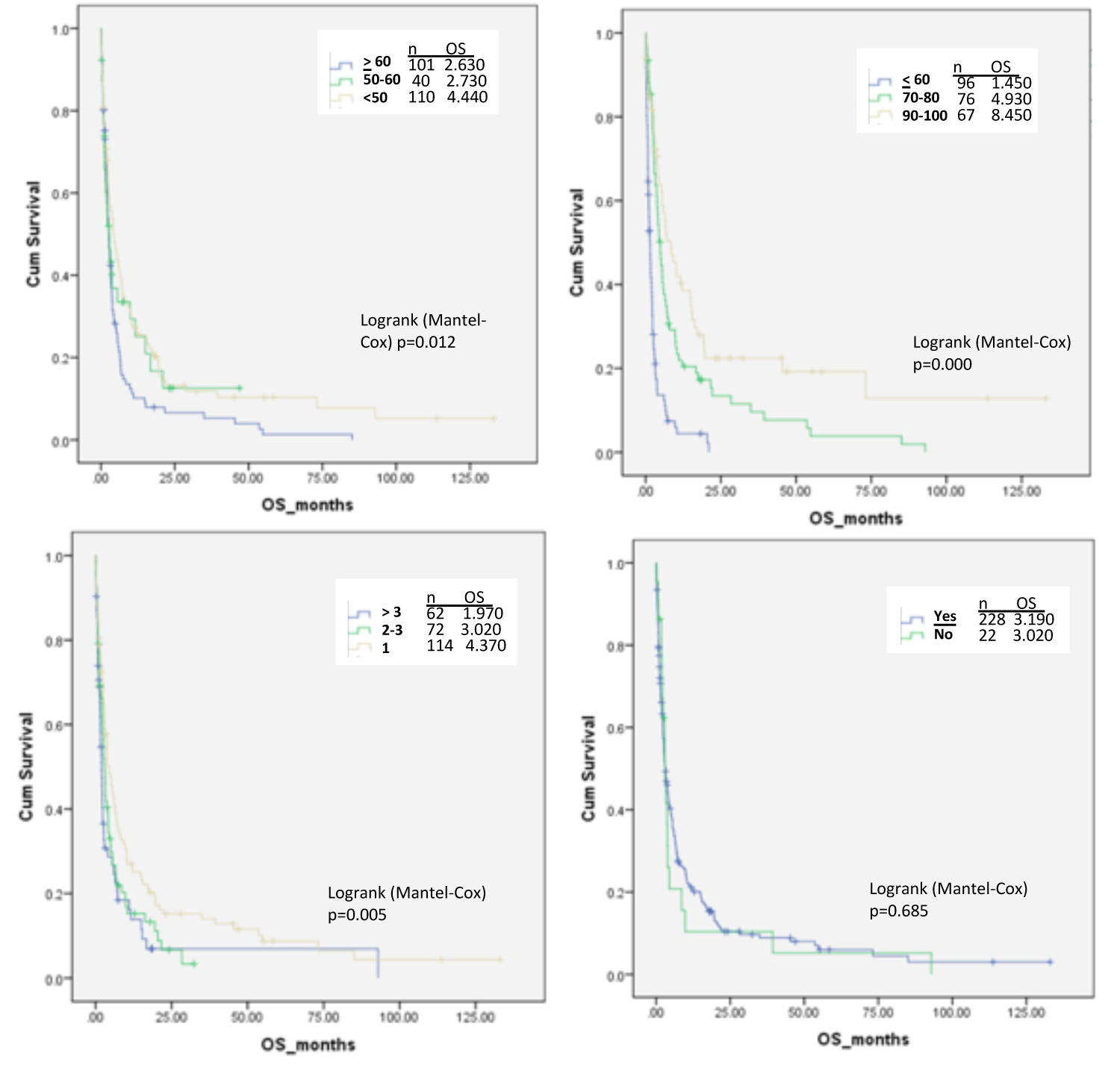

Supplement: Supplementary file 4 — Additional file 4: Figure S2. Original GPA components applied in our sarcoma cohort. A. Age; B. Karnofsky performance status (KPS); C. Number of CNS lesions; D. Presence of extracranial metastases. CNS: central nervous system; OS: overall survival. [file 12885_2020_6548_MOESM4_ESM.tif]

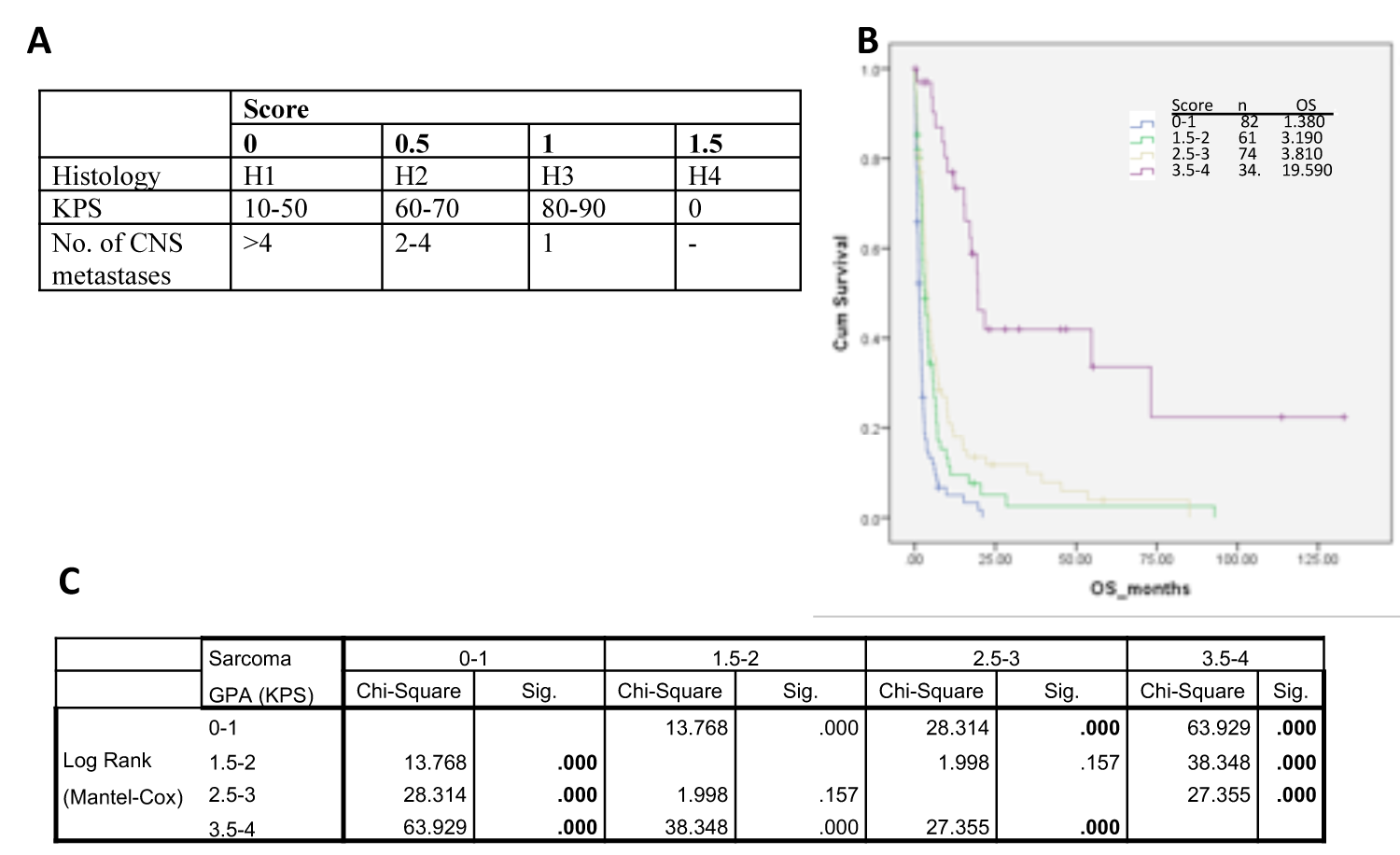

Supplement: Supplementary file 5 — Additional file 5: Figure S3. Sarcoma Graded Prognostic Assessment index based on KPS. A: Prognostic factors, point groupings and Mann-Whitney test for significance of split-levels; B: Kaplan-Meier curves for overall survival levels by Sarcoma-GPA group; C: Pairwise comparisons using the Mantel-Cox logrank test. H1-H4: histology groups (see text for description). CNS: central nervous system; KPS: Karnofsky performance status; OS: overall survival. [file 12885_2020_6548_MOESM5_ESM.tif]
